# Supplementary material for: Parallel Evolution of Pseudomonas aeruginosa during a Prolonged ICU-Infection Outbreak
Source: Microbiol Spectr. 2022 Nov 7;10(6):e02743-22. doi: 10.1128/spectrum.02743-22 (PMC9769503; doi:10.1128/spectrum.02743-22)
Supplement: Supplemental file 1 — Supplemental material. Download spectrum.02743-22-s0001.pdf, PDF file, 0.6 MB [file spectrum.02743-22-s0001.pdf]

SUPPLEMENTAL MATERIAL

Parallel evolution of *Pseudomonas aeruginosa* during a prolonged ICU infection outbreak

David R. Cameron[1,#], Melissa Pitton[1,2], Simone Oberhaensli[3], Katja Schlegel[4], Dominique S. Blanc[5], Stephan M. Jakob[1], and Yok-Ai Que[1,#]

1. Department of Intensive Care Medicine, Inselspital, Bern University Hospital, University of Bern, Bern, Switzerland

2. Graduate School for Cellular and Biomedical Sciences (GCB), University of Bern, Bern, Switzerland

3. Interfaculty Bioinformatics Unit and SIB Swiss Institute of Bioinformatics, University of Bern, Bern, Switzerland

4. Institute of Psychology, University of Bern, Bern, Switzerland

5. Service of Hospital Preventive Medicine, Lausanne University Hospital and University of Lausanne, Lausanne, Switzerland

#Corresponding authors
Yok-Ai Que, MD, PhD
Department of Intensive Care Medicine, INO E-403
Inselspital; Bern University Hospital
3010 Bern, Switzerland
Tel: +41 31 632 45 21
E-Mail: Yok-Ai.Que@insel.ch

David R. Cameron, PhD
Department of Intensive Care Medicine, INO E-403
Inselspital; Bern University Hospital
3010 Bern, Switzerland
E-Mail: davidrobert.cameron@insel.ch

Keywords: adaptative evolution, antibiotic resistance, carbapenems, *oprD*, outer membrane porin D.

INDEX

Table S1. ***Pseudomonas aeruginosa*** **isolates assessed in the current study.**

Table S2. Mutation matrix for 58 ***P. aeruginosa*** **isolates.**

Table S3. Isolate-centric list of mutations with statistics (coverage, p-values).

Table S4. PseudoCAP functional classification of each mutated gene.

Figure S1. Correlation analysis for growth performance and motility. Pearson r values and p-values were determined using GraphPad Prism.

Figure S2. Cluster quality assessment. Phenotypic data were z-transformed and used to identify clusters using the TwoStep method. The quality of the clusters received the highest grade ("good"). Mean and standard deviations for each cluster are provided.

Figure S3. Sequence read mapping statistics. Sequence reads were mapped to the complete genome of H25883 (accession: CP033686). **(A)** The number of reads that were mapped for each isolate, and the depth of coverage. **(B)** The percent of the H25883 genome that was covered by reads for each isolate, and the mean pairwise identity for the reads. Each symbol represents an independent isolate, and the data is summarised by the mean.

Figure S4. Resistant isolates across different anti-*Pseudomonal* antibiotic classes. The percentage of resistant isolates is shown across different, relevant classes of antibiotics commonly used to treat *P. aeruginosa* infections. Resistance to a class was defined as the resistance to at least one antibiotic belonging to the class. Carbapenems: meropenem and imipenem (n=39); penicillins: piperacillin/tazobactam (n=39); cephalosporins: ceftazidime and cefepime (n=39); quinolones: ciprofloxacin and levofloxacin (n=39); colistin (n=23).

Figure S5. Phenotypic data for *P. aeruginosa* from each of four clusters (classed A-D). (Panel A) Growth performance. **(B)** Motility. **(C)** Meropenem minimum inhibitory concentration (MIC). Data are summarised by the mean.

Figure S6. Additional pathoadaptive traits evolving during the ICU infection outbreak. (A) Isolates with *lasR* mutation were identified repeatedly which correlated with overproduction of blue-green pigment and colony autolysis. **(B)** A single introduction of a mucA mutation was identified which correlated with the mucoidy phenotype.

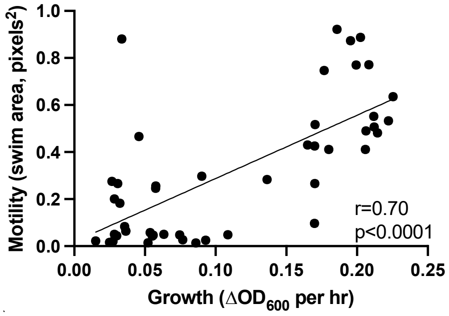

Figure S1. Correlation analysis for growth performance and motility. Pearson r values and p-values were determined using GraphPad Prism.

Model Summary

**Algorithm**	TwoStep
**Inputs**	3
**Clusters**	4

Cluster Quality

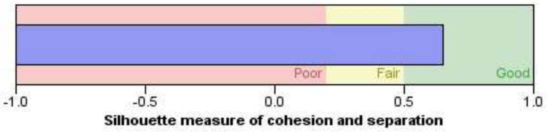

		ZOD_change		ZSwim		Zmer_mic_log	
		Mean	Std. Deviation	Mean	Std. Deviation	Mean	Std. Deviation
Cluster	A	1.1660986	0.28070449	1.0210867	0.63168202	-1.0320439	0.21880678
	B	-0.8601866	0.25280467	-0.7924384	0.31349292	-0.8618315	0.28135720
	C	0.7515887	0.74024831	0.6781965	0.97760174	0.7890471	0.52589673
	D	-0.7530285	0.38397360	-0.6426658	0.48021809	0.9842166	0.31440924
	Combined	0.0000000	1.00000000	0.0000000	1.00000000	0.0000000	1.00000000

Figure S2. Cluster quality assessment. Phenotypic data were z-transformed and used to identify clusters using the TwoStep method. The quality of the clusters received the highest grade ("good"). Mean and standard deviations for each cluster are provided.

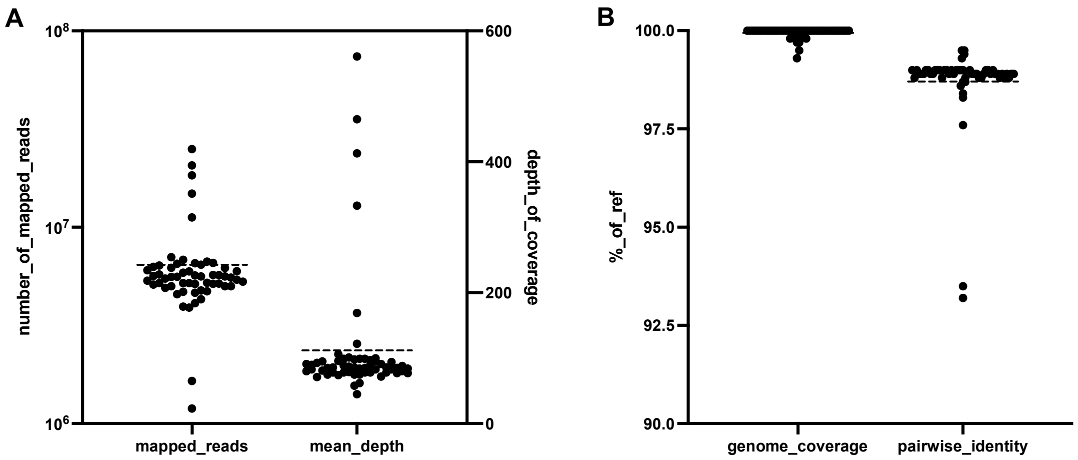

Figure S3. Sequence read mapping statistics. Sequence reads were mapped to the complete genome of H25883 (accession: CP033686). **(A)** The number of reads that were mapped for each isolate, and the depth of coverage. **(B)** The percent of the H25883 genome that was covered by reads for each isolate, and the mean pairwise identity for the reads. Each symbol represents an independent isolate, and the data is summarised by the mean.

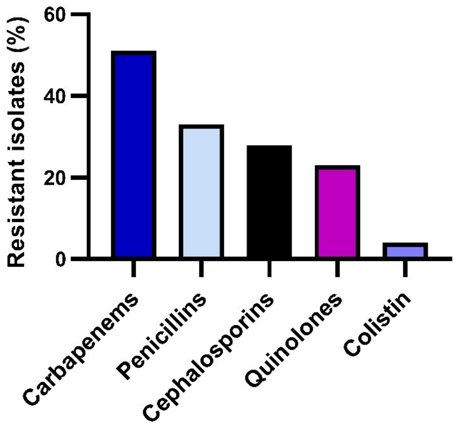

Figure S4. Resistant isolates across different anti-*Pseudomonal* antibiotic classes. The percentage of resistant isolates is shown across different, relevant classes of antibiotics commonly used to treat *P. aeruginosa* infections. Resistance to a class was defined as the resistance to at least one antibiotic belonging to the class. Carbapenems: meropenem and imipenem (n=39); penicillins: piperacillin/tazobactam (n=39); cephalosporins: ceftazidime and cefepime (n=39); quinolones: ciprofloxacin and levofloxacin (n=39); colistin (n=23).

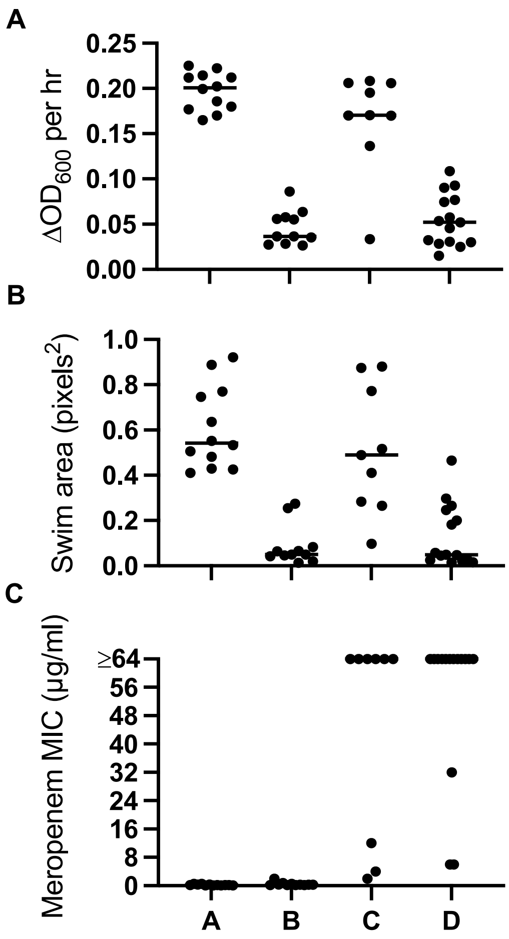

Figure S5. Phenotypic data for *P. aeruginosa* from each of four clusters (classed A-D). (Panel A) Growth performance. **(B)** Motility. **(C)** Meropenem minimum inhibitory concentration (MIC). Data are summarised by the mean.

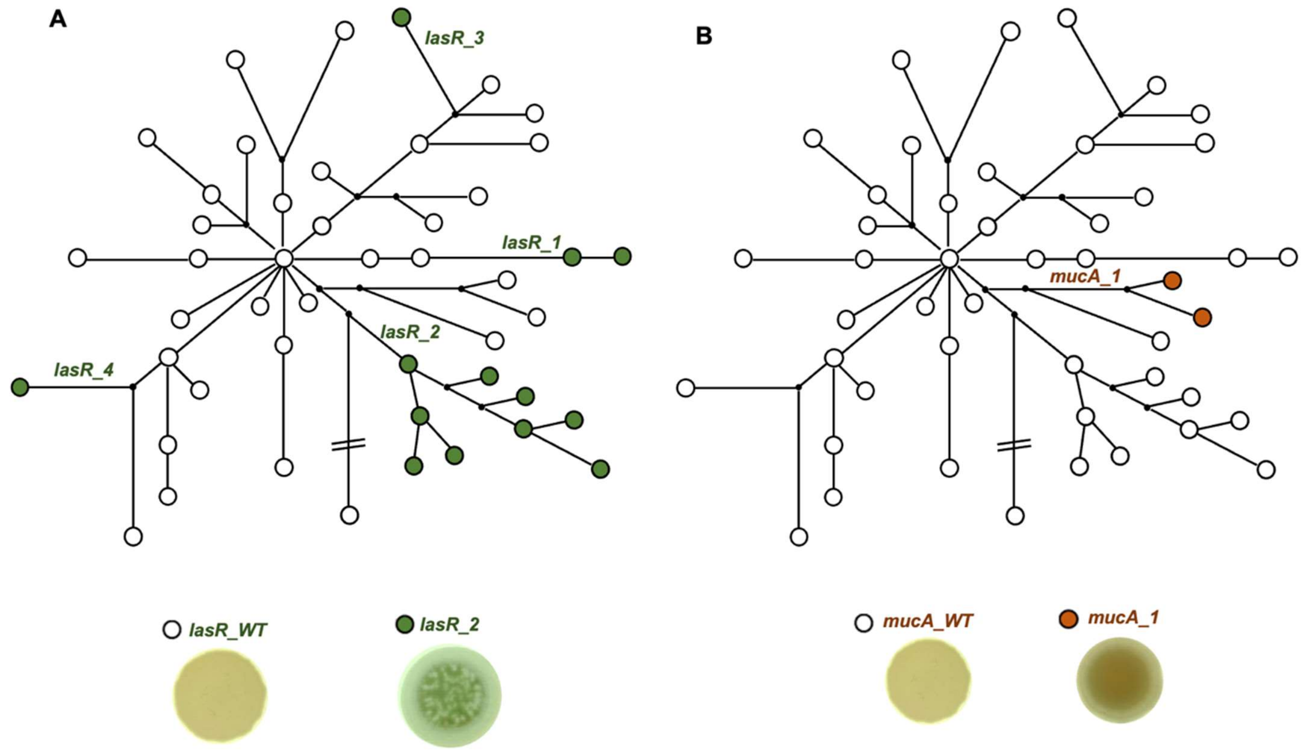

Figure S6. Additional pathoadaptive traits evolving during the ICU infection outbreak. (A) Isolates with *lasR* mutation were identified repeatedly (n=4 independent mutations) which correlated with overproduction of blue-green pigment and colony autolysis. **(B)** A single introduction of *mucA* mutation was identified which correlated with the mucoidy phenotype.
